# A New Tool for Safety Evaluation and a Combination of Measures for Efficacy Assessment of Cotransplanting Human Allogenic Neuronal Stem Cells and Mesenchymal Stem Cells for the Treatment of Parkinson Disease: Protocol for an Interventional Study

**DOI:** 10.2196/29695

**Published:** 2021-10-22

**Authors:** Fatima Jamali, Mayis Aldughmi, Mohammad W Khasawneh, Said Dahbour, Alaa A Salameh, Abdalla Awidi

**Affiliations:** 1 Cell Therapy Center University of Jordan Amman Jordan; 2 Department of Physiotherapy School of Rehabilitation Sciences University of Jordan Amman Jordan; 3 Medical Faculty University of Jordan Amman Jordan; 4 Neurology Division Jordan University Hospital Amman Jordan; 5 Hematology Department Jordan University Hospital Amman Jordan

**Keywords:** Parkinson disease, neurodegenerative disease, regenerative medicine, mesenchymal stem cells, MSCs, neuronal stem cells, NSCs, Unified Parkinson Disease Rating Scale, UPDRS, Mobility Lab, α-synuclein, PARK-7, stem cells, stem cell therapy, therapeutics, Parkinson’s, neurological diseases

## Abstract

**Background:**

Parkinson disease (PD) is a neurodegenerative disorder associated with a broad spectrum of motor and nonmotor symptoms. Any proposed cure needs to address the many aspects of the disease. Stem cell therapy may have potential in this regard as indicated in recent preclinical and clinical studies.

**Objective:**

This protocol aims to examine the safety and therapeutic benefit of human Wharton jelly-derived mesenchymal stem cells (WJ-MScs) and their derivatives, neuronal stem cells (NSCs) in PD.

**Methods:**

This clinical trial is a double-arm, single-blinded, phase I-II interventional study. Participants have been allocated to 1 of 2 groups: one receiving allogeneic WJ-MSCs alone, the other receiving NSCs and WJ-MScs. Participants are being followed-up and assessed over a period of 6 months. To assess safety, an incidence of treatment-emergent adverse events (TEAEs) tool tailored for PD is being used immediately and up to 6 months after treatment. For efficacy assessment, a number of factors are being used, including the gold standard severity test and the Unified Parkinson Disease Rating Scale. In addition, the following standardized assessments for different common symptoms in PD are being included: motor (both subjectively and objectively assessed with wearable sensors), sensory, quality of life and psychological well-being, cognition, and sleep quality. Furthermore, immune-modulatory cytokines and neuronal damage versus regeneration markers in PD, including the neuronal protein linked to PD, α-synuclein, are being monitored.

**Results:**

Ten patients have been enrolled in this study and thus participant recruitment has been completed. The study status is active and beyond the recruiting stage. Study chart implementation, data collection, and analysis are ongoing.

**Conclusions:**

The combination of NSCs and MSCs in PD may be useful for harnessing the best of the immunomodulation and neural repair characteristics of these cell types. The tailored comprehensive and scaled TEAEs and the variety of evaluation tools used enables a comprehensive assessment of this cellular therapy treatment protocol. A consideration of this expanded tool set is important in the design of future clinical studies for PD.

**Trial Registration:**

ClinicalTrials.gov NCT03684122; https://clinicaltrials.gov/ct2/show/NCT03684122

**International Registered Report Identifier (IRRID):**

DERR1-10.2196/29695

## Introduction

Parkinson disease (PD) is the most common movement disorder and the second most common neurodegenerative disorder of aging [1]. This neurodegenerative disease arises due to the loss of dopaminergic neurons of the substantia nigra pars compacta, a basal ganglia structure located in the midbrain that plays an important role in bodily movement. PD is commonly recognized through clinical motor signs of tremor, rigidity, and bradykinesia, with postural instability often appearing as the disease progresses. Nevertheless, PD is associated with a broad spectrum of nonmotor symptoms that habitually precede motor symptoms and that have been found to be associated with a reduced quality of life [2]. These signs include mood disorders, cognitive dysfunction, sensory dysfunction with hyposmia, and disturbances of sleep-wake cycle regulation [3].

Conventional treatment strategies for managing motor and nonmotor symptoms of PD have included medical and surgical methods. Medical therapy relies on replenishing dopaminergic activity in the basal ganglia, with levodopa being the pillar of the medical therapy. In addition, deep brain stimulation is the most common surgical procedure and is designed to mitigate the motor symptoms of PD by targeting the subthalamic nucleus and globus pallidus par interna [4]. Although these therapies are helpful in alleviating and halting the symptoms in many cases, some patients do not respond to these methods while others suffer their side effects. In levodopa-based medical therapy, the drug effect usually wears off after a short time as the disease progresses, requiring increased frequency of dosing [5]. Moreover, even though deep brain stimulation has been approved by the US Food and Drug Administration for PD treatment, its related adverse events are unpredictable [6]. They may include hardware-related complications leading to infection and stimulation-induced phenomena, such as paresthesia, dysarthria, ataxia, and hypotonia.

In recent years, regenerative medicine has been investigated in neurological diseases, including PD, with a clear potential being demonstrated but at varying efficacy levels. Several types of stem cells have been used in experimental studies related to PD, including embryonic pluripotent stem cells, mesenchymal stem cells (MSCs), and induced pluripotent stem cells. MSCs have been studied extensively and present several advantages over other types of stem cells. These include minimum manipulation, relatively high genetic stability, and ease of isolation and accessibility from various tissues, such as bone marrow, adipose tissue, and peripheral blood. The umbilical cord is the most accessible source of MSCs than can be used to generate large numbers of high proliferating human Wharton jelly-derived MSCs (WJ-MSCs) which can be stored in biobanks. MSCs have been found to be capable of replacing and rescuing degenerated dopaminergic and nondopaminergic neurons, suggesting its potential for the treatment of both motor and nonmotor symptoms of PD [7].

The multipotency characteristic of MSCs enables them to differentiate into many cell types, including neurons and other neuronal cells [8]. WJ-MSCs differentiated into neuronal stem cells (NSCs) were found to have enhanced therapeutic potential compared to WJ-MSCs. NSCs exhibit neuroectodermal characteristics with reduced capacity to undergo mesodermal differentiation while preserving their immune modulatory properties [9]. This means that NSCs are more committed to the neuroectodermal lineage with minimized risk of ectopic differentiation following central nervous system transplantation [10].

The safety and clinical outcome of using allogenic MSCs alone or alongside their derived NSCs in PD individuals have not yet been investigated. The available research on the safety and efficacy of therapeutic agents for PD is rarely comprehensive because not many aspects of the disease are examined, leading to an incomplete evaluation. Therefore, this study has been designed to assess and compare NSCs cotransplanted with MSCs to MSCs alone. The study’s protocol aims to produce clear conclusions regarding the safety and the comparative benefits of both treatment arms in regard to the different aspects of life commonly affected by PD.

## Methods

### Ethical Approval and Recruitment

The study protocol was submitted to the Research Ethics Committee of the Cell Therapy Center (CTC) at the University of Jordan (approval #IRB/01/2018). All study procedures are following the ethical principles of the Helsinki declaration. Trained research personnel explained benefits and risks of participation in this study during the consent process, and all eligible participants signed an informed consent form. The study protocol is registered in the American Registry of Clinical Trials (NCT03684122). 

Potential participants were recruited using study flyers that were distributed in the neurology clinic at Jordan University Hospital, shared on different social media platforms and the CTC official website. Flyers included an invitation for interested people with PD to participate in the study, a brief explanation of the aims and design of the study, and contact numbers of study team members responsible for participants’ recruitment. Subsequent phone interviews were conducted with interested individuals. Screening information collected included participant’s profile, past medical history, current medication list, date of diagnosis with PD, and current functional limitations. Consequently, participants were scheduled for their baseline assessment, in which they were considered as eligible or ineligible according to the predefined criteria regardless of gender, ethnicity, or social background.

### Study Design

**Figure 1:**
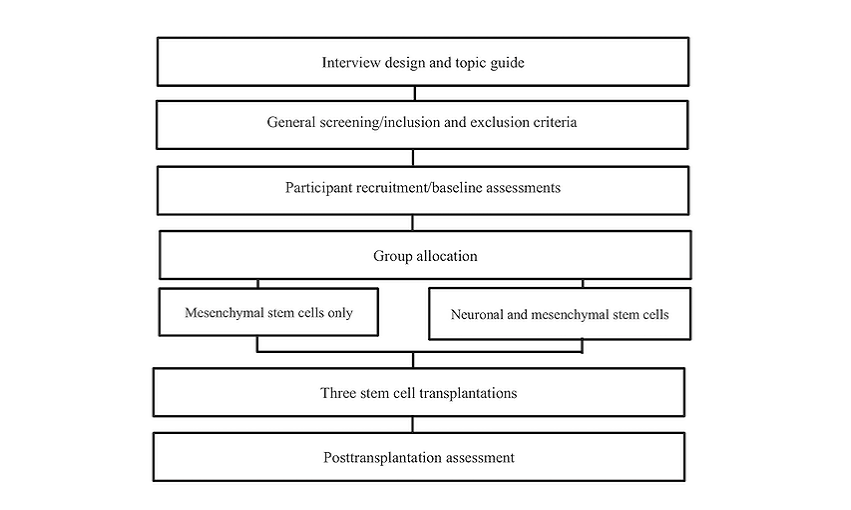
Study plan summarizing the different stages of the clinical trial.

This study is a double-arm, single-blinded, phase I/-II interventional clinical trial. The protocol is designed to compare injecting MSCs and their derived NSCs to injecting MSCs alone. Ten participants with PD who matched the inclusion and exclusion criteria detailed in the next section were assigned into 1 of 2 arms. Participants in the 2 groups were matched based on age, gender, disease severity (according to the Hoehn and Yahr Scale) and Unified Parkinson Disease Rating Scale (UPDRS) scores. All participants are being assessed at 3 different time points: up to 1 week before first intervention, and at 3 months and 6 months after. The study design is summarized in Figure 1.

The primary objective is to evaluate the safety of the cellular therapy protocols. Immediate, short-term, and up-to-6-month treatment-emergent adverse events (TEAEs) are being thoroughly examined. The secondary outcome includes the efficacy of both stem cell therapy protocols which will evaluated by documenting and correlating changes in motor and nonmotor functions, in addition to examining biological markers of inflammation and neural regeneration and repair.

### Inclusion and Exclusion Criteria

Participants were required to meet the following inclusion criteria in order to be included in the study: nonsmoker; aged between 18 and 80 years; physician diagnosis of idiopathic PD confirmed from a medical file provided by participants; disease duration between 1 and 15 years; robust response to dopaminergic therapy (defined as greater than 33% reduction in symptoms on the UPDRS when measured in the “On medicine” state compared to the “Off medicine” state); for participants taking any central nervous system–related medications (eg, benzodiazepines, antidepressants, hypnotics), a regimen that is optimized and stable for 90 days prior to the screening visit; stable PD symptomatic therapy for at least 90 days prior to screening; for women of childbearing potential, a reliable form of contraception from 30 days prior to baseline visit until 6 months after treatment; and a clear infectious panel examination including hepatitis B and C, HIV, and syphilis.

Meanwhile, if participants met any of the following exclusion criteria, they were deemed ineligible to participate in the study: a typical or drug-induced Parkinsonism; a UPDRS rest tremor score of 3 or greater for any limb on medication; a Montreal Cognitive Assessment score of less than 25; clinical features of psychosis or refractory hallucinations, uncontrolled seizure disorder, defined as a seizure within the last 6 months; developmental delay; hepatic disease or altered liver function as defined by alanine transaminase >150 U/L and or T bilirubin >1.6 mg/dl at admission; history of congestive heart failure or clinically significant bradycardia and presence of second- or third- degree atrioventricular block; active malignancy or diagnosis of malignancy within 5 years prior to the start of screening (with cancer-free status for at least 5 years being permitted, and skin cancers, except for melanoma, being permitted); history of stroke or traumatic brain injury; major surgery within the previous 3 months or planned in the ensuing 6 months’ clinically significant abnormalities in the screening laboratory studies; history of use of an investigational drug within 30 days prior to the screening visit; unable to return for follow-up visits for clinical evaluation and laboratory studies; and any other condition the investigator feels would pose a significant hazard to the participant if enrolled or that would complicate the study assessments.

### Baseline Assessments

Participants were scheduled for an initial evaluation at the CTC, University of Jordan. Participants were then asked to fill and sign a consent form, which was explained thoroughly by research personnel. Thereafter, participants underwent a comprehensive battery of measures including motor and nonmotor medical assessments, which took approximately 3 hours to complete. By the end of the baseline assessment, it was determined whether a participant was eligible for inclusion or not. All participants deemed eligible were scheduled to undergo posttreatment assessments after 3 and 6 months.

### Stem Cell Preparation

#### Human WJ-MSCs

Stem cell injections were prepared from thawed WJ-MSCs previously cryopreserved in the biobank of the CTC. Isolation of MSCs from healthy and bioscreened umbilical cord donor was performed according to good laboratory practice standard operation protocols. Characterization of the isolated stem cells was required to meet the defining criteria of MSCs, including microscopic spindle shape, surface marker expression, and trilineage differentiation, in accordance with the International Society for Cellular Therapy recommendations [11]. Briefly, the cord was rinsed with phosphate-buffered saline (PBS; pH 7.4) and cut into 5-cm-long pieces. The pieces were cut longitudinally, and the blood vessels were removed. The remaining tissues were cut into ~4 mm^2^ pieces and plated in tissue culture plates. The explants were allowed to attach for about 15 minutes, and then culture medium was gently added to the plates and incubated without moving for 8 days.

The WJ-MSCs’ expansion culture medium consisted of α-modified minimum essential medium (Thermo Fisher Scientific) supplemented with 5 % human platelet lysate, 1% (w/v) penicillin/streptomycin, and 2 mM of L-glutamine (Thermo Fisher Scientific).

Subsequently, culture medium was changed every 3 to 4 days, and cells were passaged at 80% confluence with xenogenic-free TrypLE 10X dissociation reagent (Thermo Fisher Scientific). Prior to cryopreservation, MSC batches were karyotyped to examine gross chromosomal aberrations. In addition, deep cytogenetic analysis was performed with Cytoscan microchips and ChAS software (Thermo Fisher Scientific).

The final cell suspensions, ranging from 80 to 120 × 10^6^ MSCs, was prepared for intrathecal injection while another of 40 to 60 × 10^6^ MSCs was prepared for intravenous injection in preservative-free normal saline. An automated cell counting instrument with disposable chambers was used to determine the number of cells administered (Thermo Fisher Scientific). The system identified cell viability and cell concentrations from 1 × 10^4^ to 1 × 10^7 ^cells per milliliter with a mean diameter detection of 5 μm to 60 μm.

#### Human Neuronal Stem Cells

The same biological sample of umbilical cord–derived MSCs was used for NSC generation. WJ-MSCs were thawed and expanded for 4 days in low-adherence flasks. The differentiation culture medium was the Neurobasal Medium (Thermo Fisher Scientific) supplemented with 2% B27 growth factor (Thermo Fisher Scientific). Cells were harvested 8 to 12 days after differentiation. Floating “neurospheres” became visible in the culture after 2 to 5 days. Immunocytochemistry staining with nestin-2 and PAX-6 was performed for further characterization of neuronal differentiation. To obtain single-cell suspensions of NSCs, neurospheres were triturated in TrypLE (10×) with a serological pipette and then diluted with PBS (Thermo Fisher Scientific). NSC genetic stability was examined after DNA extraction (Qiagen) and compared to thawed MSCs using Cytoscan microchips and ChAS software (Thermo Fisher Scientific).

Prior to injection, cells were centrifuged, resuspended with PBS for counting and diluted to the final cell number between 8 and 12 × 10^6^ NSCs per injection in preservative-free normal saline. Cells were delivered to the clinical unit in the CTC for immediate use.

### Stem Cell Transplantation Protocol

**Figure 2:**
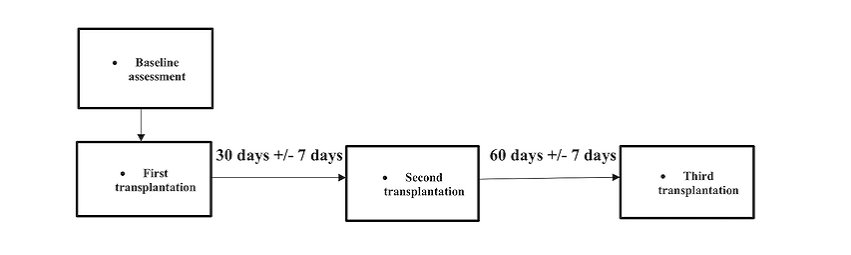
Stem cell transplantation schedule.

Stem cell transplantation for each participant was held at the ICU rooms of the CTC clinical unit. Vital signs of each participant were monitored prior and throughout the procedure. The treatment protocol entails 3 consecutive injections: the first within a week of the baseline assessment, and the second and third 1 month and 3 months after, respectively (Figure 2).

At each time point, MSCs were intravenously delivered for all patients and either NSCs or MSCs were cotransplanted intrathecally. For intrathecal injections, 1 ml to 2 ml of 2% lidocaine hydrochloride solution, a local anesthetic agent, was used. Spine X-ray images were examined by the physician to take into consideration any anatomical variations. A disposable, sterilized epidural kit was used for every participant with appropriate long-needle size according to participant’s weight. A volume of 5 ml to 10 ml of cerebrospinal fluid was aspirated and immediately frozen at –80 °C, followed by stem cell transplantation of a similar volume to limit central nervous system pressure with consequent headaches.

### Treatment-Emergent Adverse Event Reporting

Preceding the intervention, vital signs measurements and extensive medical history with complete system review for enrolled participants were performed by trained nursing staff in the CTC.

Following the intervention, TEAEs were monitored and recorded according to the following schedule: immediate follow-up, 24-hour follow-up, 1-week follow-up, 1-month follow-up, and 3-month follow-up. Based on available TEAE assessment tools, a detailed scaled questionnaire was compiled specifically for this trial [12-14]. This enables a comprehensive assessment of any relevant symptoms that might appear after administration of a new treatment regimen for PD and is designed to be used for related neurological diseases (Table 1).

**Table 1:** Scaled incidence of treatment-emergent adverse event (TEAEs) reporting tool for immediate assessment.

Adverse effect	Grade				
	1	2	3	4	5
Allergy	Transient flushing or rash; drug fever <38 °C (<100.4 °F)	Rash; flushing; urticaria; dyspnea; drug fever ≥38 °C (≥100.4 °F)	Symptomatic bronchospasm, with or without urticaria; parenteral medication(s) indicated; allergy-related edema or angioedema; hypotension	Anaphylaxis	Death
Pain	Mild pain not interfering with function	Moderate pain; pain or analgesics interfering with function, but not interfering with ADL^a^	Severe pain; pain or analgesics severely interfering with ADL	Disabling	N/A^b^
Injection site swelling	<2.5 cm	2.5-5 cm	>5 cm	N/A	N/A
Rash	Macular or papular eruption or erythema without associated symptoms	Macular or papular eruption or erythema with pruritus or other associated symptoms; localized desquamation or other lesions covering <50% of BSA^c^	Severe and generalized erythroderma, macular, papular, or vesicular eruption; desquamation covering ≥50% BSA	Generalized exfoliative, ulcerative, or bullous dermatitis	Death
Vasovagal episode	N/A	Present without loss of consciousness	Present with loss of consciousness	Life-threatening consequences	Death
Dizziness	With head movements or nystagmus only; not interfering with function	Interfering with function, but not interfering with ADL	Interfering with function, but not interfering with ADL	Disabling	N/A
Fever	38.0-39.0 °C (100.4-102.2 °F)	>39.0-40.0 °C (102.3-104.0 °F)	>40.0°C (>104.0 °F) for ≤24 hours	>40.0 °C (>104.0 °F) for >24 hours	Death
Palpitation	Present	Present with associated symptoms (eg, lightheadedness, shortness of breath)	N/A	N/A	N/A
Numbness	Mild symptoms	Mild symptoms	Severe symptoms; limiting self-care ADL	N/A	N/A
Speech impairment	N/A	Awareness of receptive or expressive dysphasia, not impairing ability to communicate	Receptive or expressive dysphasia, impairing ability to communicate	Inability to communicate	N/A
Tremor	Mild and brief or intermittent but not interfering with function	Moderate tremor interfering with function, but not interfering with ADL	Severe tremor interfering with ADL	Disabling	N/A
Muscle cramp/soreness	Mild pain	Moderate pain limiting instrumental activities of daily living	Severe pain limiting self-care activities of daily living	N/A	N/A
Seizures	N/A	One brief generalized seizure; seizure(s) well controlled by anticonvulsants or infrequent focal motor seizures not interfering with ADL	Seizures in which consciousness is altered; poorly controlled seizure disorder, with breakthrough of generalized seizures despite medical intervention	Seizures of any kind which are prolonged, repetitive, or difficult to control (eg, status epilepticus, intractable epilepsy)	Death
Fatigue	Mild fatigue over baseline	Moderate or causing difficulty in performing some ADL	Severe fatigue interfering with ADL	Disabling	N/A
Blurred vision	Symptomatic and not interfering with function	Symptomatic and interfering with function, but not interfering with ADL	Symptomatic and interfering with ADL	Disabling	N/A
Sweating	Mild and occasional	Frequent or drenching	N/A	N/A	N/A
Fecal incontinence	Occasional use of pads required	Daily use of pads required	Severe symptoms; elective operative intervention indicated	N/A	N/A
Blood with stool	Mild symptoms; intervention not indicated	Moderate symptoms; intervention indicated	Transfusion indicated; invasive intervention indicated; hospitalization	Life-threatening consequences; urgent intervention indicated	Death
Anorexia	Loss of appetite without alteration in eating habits	Oral intake altered without significant weight loss or malnutrition; oral nutritional supplements indicated	Associated with significant weight loss or malnutrition (eg, inadequate oral caloric and/or fluid intake); tube feeding or TPN^d^ indicated	Life-threatening consequences; urgent intervention indicated	Death
Nausea	Loss of appetite without alteration in eating habits	Oral intake decreased without significant weight loss, dehydration or malnutrition	Inadequate oral caloric or fluid intake; tube feeding, TPN, or hospitalization indicated	N/A	N/A
Vomiting	1 episode in 24 hours	2-5 episodes in 24 hours; IV^e^ fluids indicated <24 hours	≥6 episodes in 24 hours; IV fluids or TPN indicated ≥24 hours	Life-threatening consequences	Death
Dry mouth	Symptomatic (eg, dry or thick saliva) without significant dietary alteration; unstimulated saliva flow >0.2 ml/min	Moderate symptoms: oral intake alterations (eg, copious water, other lubricants, diet limited to purees and/or soft, moist foods); unstimulated saliva 0.1-0.2 ml/min	Inability to adequately aliment orally; tube feeding or TPN indicated; unstimulated saliva	N/A	N/A
Urine incontinence	Occasional (eg, with coughing, sneezing, etc); pads not indicated	Spontaneous; pads indicated	Interfering with activities of daily living; intervention indicated (eg, clamp, collagen injections)	Operative intervention indicated (eg, cystectomy or permanent urinary diversion)	N/A

^a^ADL: ability to perform activities of daily living.

^b^N/A: not applicable.

^c^BSA: body surface area.

^d^TPN: total parenteral nutrition.

^e^IV: intravenous.

### Follow-up Assessments of Common PD Symptoms

**Figure 3:**
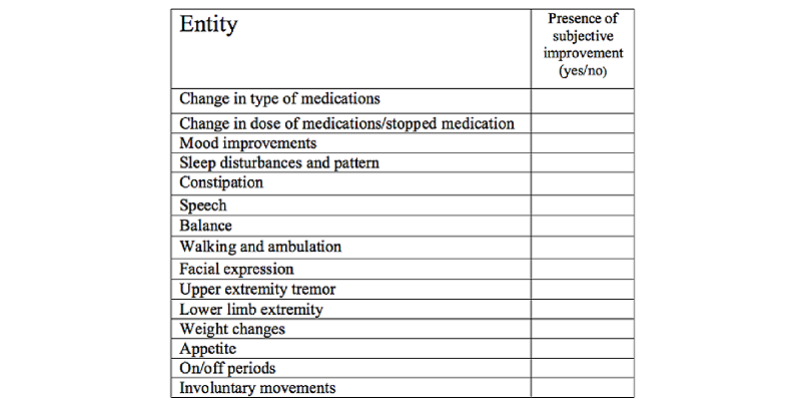
One-month posttransplantation subjective evaluation questionnaire.

A detailed follow-up of the different common manifestations of the disease and quality of life were examined at 3- and 6-month postinjection and then compared to baseline assessments. In addition, an over-the-phone subjective evaluation was performed 1 month after the first transplantation, in which a predesigned questionnaire was used to report any changes perceived by patients (Figure 3). The components of follow-up assessment are detailed in the following sections.

### Disease Severity Assessments

#### Unified Parkinson Disease Rating Scale

The UPDRS serves as a disability and impairment scale for progression follow-up. It assesses changes in overall function and is divided into 4 sections: (1): evaluation of mentation, behavior, and mood (13-item assessment); (2) evaluation of activities of daily living, including speech, swallowing, handwriting, dressing, hygiene, falling, salivation, turning in bed, and wasting and cutting food (13-item assessment); (3) motor examination (18-item assessment of the right and left sides, and other body functions such as speech); and (4) motor complications (6-item assessment)

#### Unified Dyskinesia Rating Scale

The Unified Dyskinesia Rating Scale [15] evaluates involuntary movements or dyskinesia often associated with PD. There are 2 primary parts: (1) historical (part 1: on-dyskinesia; part 2: off-dystonia) and (2) objective (part 3: impairment; part 4: disability).** **

### Motor and Functional Mobility Assessments

#### Mobility Lab

Mobility Lab [16] (APDM Inc) is a wearable mobility system that objectively assesses gait and balance, and has been validated for use in the PD population. It involves wearing wireless sensors on the wrists, ankles, and trunk. The participants wear the sensors while performing walking and turning tasks. Data are analyzed using the APDM software.

#### Nine-Hole Peg Test

The Nine-Hole Peg Test [17] is a specific test for finger and manual dexterity. Participants are asked to pick up 9 pegs one at a time from a container, using 1 hand only, and put them into holes as quickly as they can one at a time. They are required, without pausing, to remove the pegs one at a time and return them to the container as quickly as they can. Time taken to finish the task from the dominant and nondominant hands is used as the outcome measure. 

#### Arabic Version of the Berg Balance Scale

The Berg Balance Scale (Arabic version) [18] is designed to objectively assess balance through 14 common functional activities that occur in everyday life. A higher score indicates a higher level of function.

#### Arabic Version of Activities-Specific Balance Confidence Scale

The Activities-Specific Balance Confidence Scale (Arabic version) [19] is a subjective measure of confidence in maintaining balance while performing various ambulatory and functional daily activities without falling or experiencing a sense of unsteadiness. A higher score indicates high confidence in maintaining balance.

#### Arabic Version of Falls Efficacy Scale-International

The Falls Efficacy Scale-International questionnaire (Arabic version) [20] is designed to assess the fear of falling in the older population and is recommended for use in people with PD. A higher score indicates that the person has a fear of falling. A lower score indicates high confidence in maintaining balance.

#### The Falls Efficacy Scale-International

For this assessment, each participant was asked to provide information about their history of falls at baseline, specifically at 1-month and 6-months pre-enrollment. In addition, information regarding the mechanism of falling was documented. Participants were contacted by research personnel every month postbaseline up to 6 months via phone to document falls.

### Sensory Assessments 

#### The University of Pennsylvania Smell Identification Test

The University of Pennsylvania Smell Identification Test [21] is commercially available for smell identification to test the function of an individual's olfactory system. It is the gold standard assessment of olfactory function among the smell identification tests for its high reliability (*r*=0.94) and practicality.

#### Arabic Version of the Pain Rating Scale

The Pain Rating Scale (Arabic version) [22] includes the visual analogue pain scale and assess different aspects of pain, including pain felt at the moment and pain felt in the past week. The Pain Rating Scale was translated by the British Pain Society and is recommended for use in populations for whom pain is a major issue.

### Quality of Life and Psychological Well-being Assessments

#### Parkinson Disease Questionnaire

The Parkinson Disease Questionnaire [23] is a quality-of-life questionnaire designed to assess how often people with PD experience difficulties across 8 different criteria.

#### Arabic Version of Beck’s Depression Inventory II

Beck’s Depression Inventory II (Arabic version) [24] is a subjective measure of how depression manifests in behavior. It not only identifies varying levels of depression but is also able to indicate changes in depression and intensity of depression over a period of time. A high score indicates higher levels of depression.

#### Arabic Version of the Modified Fatigue Impact Scale

The Modified Fatigue Impact Scale (Arabic version) [25] is a self-reported measure that assess the effects of fatigue in terms of physical, cognitive, and psychosocial functioning. A high score indicates higher levels of fatigue.

### Cognitive Function Assessments

#### Montreal Cognitive Assessment

Montreal Cognitive Assessment [26] is a screening assessment tool for global cognitive function. It assesses several cognitive domains. A cutoff score of 25 is indicative of mild cognitive impairment.

#### Arabic Version of the Stroop Test

The Stroop Test (Arabic version) [27] is a measure of executive function that requires participants to inhibit the natural response (reading the letter X or a color word) and replace it with another response (the color of the letter X or the color of the word)**.** Correct responses in 45 seconds are being used as the outcome measure.

#### The Symbol Digit Modalities Test

The Symbol Digit Modalities Test [28] is a measure of information-processing speed that requires participants to quickly say the number that matches a corresponding symbol. The total correct responses in 90 seconds are used as the outcome measure. 

### Sleep Quality Assessments

#### Arabic Version of the Pittsburgh Sleep Quality Index

Pittsburgh Sleep Quality Index (Arabic version) [29] is a well-validated and reliable measure of global sleep quality. It consists of 8 questions scored based on yes-or-no answers. If 3 or more items are answered yes, the person is at a high risk for obstructive sleep apnea.

#### Arabic Version of the Epworth Sleepiness Scale

The Epworth Sleepiness Scale (Arabic version) [30] is used to assess daytime sleepiness. It consists of 8 assessment items in which the participants use a 4-point Likert scale to rate how likely they would fall asleep in 8 different scenarios of daily activities. A score > 10 indicates the presence of daytime sleepiness.

#### Arabic Version of the STOP-Bang Questionnaire

The of the STOP-Bang Questionnaire (Arabic version) [31] is a simple and reliable diagnostic tool to screen participants at risk of obstructive sleep apnea.** **It consists of 8 questions scored based on yes-or-no answers. If 3 or more items are answered yes, the person is at a high risk for obstructive sleep apnea.

#### Arabic Version of the Insomnia Severity Index

The Insomnia Severity Index (Arabic version) [32] is a tool designed to assess the severity of both nighttime and daytime components of insomnia. It consists of 7 questions, and a score ≥10 is indicative of clinical insomnia.

### Immune Modulation and Neural-Regeneration Biomarkers

A combination of indicators in the blood and cerebrospinal fluid are being examined to monitor treatment-related changes in the 2 treatment groups compared to baseline. This is necessary due to the lack of consensus on a single PD progression biomarker. For this, blood and cerebrospinal fluid samples at 3 time points were rapidly separated into single-use aliquots and frozen in –80 C^0^ refrigerators at the same facility, thus ensuring sample quality is preserved until the end of the clinical trial. Two sets of multiplex enzyme-linked immunoassay kits were used on the Luminex 200 platform (Luminex Corporation) at CTC’s proteomics laboratory according to the manufacturer’s recommendations [33]. The first kit detects protein levels of α-synuclein and DJ-1/PD 7 (linked to neurodegeneration, as well as a proposed cognitive impairment marker, epidermal growth factor) [34,35]. Proinflammatory cytokines produced in the brain and peripheral blood are considered important cues in the disease. Therefore, the second kit will measure cytokine protein levels, including interleukin 1 beta, interleukin 6, interleukin 2, and tumor necrosis factor alpha [36,37].

In order to limit cross-batch variation, baseline, and 3- and 6-month posttreatment samples of each participant are being run on the same plate.

### Statistical Analysis

For data analysis, SPSS 23.0 (IBM Corporation) will be used to perform all statistical analyses, with α=.05. A 2-sided, independent *t* test will be used to test the differences between the 2 study groups at baseline. Repeated measures analysis of variance will be used to test the effect of the intervention at different time points. Pearson correlation coefficient will be used to assess the relationship between the outcome measures of interest. Biomarker analysis will be performed using the Mann-Whitney statistical test. 

### Participant Withdrawal

According to the Helsinki declaration, participants have the right to withdraw from the study at any time and are informed of this right during the written informed consent process and during the study.

### Data Management and Quality Control

The principal investigator, coinvestigators, research assistants, research coordinators, and medical doctors and nurses involved will ensure the good conduct of the data collection, data entry, and storage of relevant data.

All members of the research team will the protect the privacy of participants and the confidentiality of data. Personal identifiers have been limited on data collection forms, and all data files are being kept in a locked file cabinet. Data will be available only to appropriate members of the research team. Each participant has been given a unique identifier (symbol and number), and their electronic data are being stored in university network drives, with data collection occurring on an encrypted password-protected laptop computer.

To ensure quality control, blood samples will be analyzed in triplicate, and data entry will be cross-checked by a second person. All devices used for the biochemical testing and all subjective assessment tools included in this study have been validated. For biomarkers, samples are being measured in triplicate with the needed controls and standards included.

## Results

The study protocol was approved by the institutional review board of the CTC on March 3, 2018. The study is being funded by the Deanship of Scientific Research at the University of Jordan. As of July 1, 2019, the date of the first patient accrual, 10 patients were enrolled in this study and thus participant recruitment has been completed. As of July 1, 2021, the status of the study is active and not recruiting. Study flow chart implementation, data collection, and data analysis are in progress. The study is estimated to be completed in November 2021.

## Discussion

PD is a neurodegenerative disease which imposes a socioeconomic burden on individuals and their relatives. Cellular therapy has proven its potential in several preclinical and clinical trials conducted in the past decades. A few studies have investigated the effect of MSCs on PD animal models and human participants. In one preclinical study, Jinfeng et al [38] showed that some PD symptoms were improved when umbilical cord–derived transduced MSCs were transplanted into a PD mice model. Stiffness of the limbs, unsteady gait, and uncoordinated limb movements were all alleviated after the stem cell treatment. Another study by Shetty et al [39] indicated that bone marrow–derived MSCs can be transdifferentiated adequately into functional dopaminergic neurons both in vitro and in vivo.

This protocol adopts a novel approach to PD cell–based therapy treatment. The novelty arises from 4 major points. The first is the choice and combination of stem cells. Umbilical cord–derived WJ-MSCs were chosen over other types of MSCs based on our earlier findings, which demonstrated an inherent advantage at the transcriptome level of this source of MSCs in terms of neurogenesis [40]. Cotransplanting allogenic NSCs in 1 arm is a unique aspect of the study, as the safety and effect of allogenic NSCs on PD symptoms have not been previously investigated. However, Harris et al [41] did pioneer the clinical use of human NSCs in a neuroinflammatory disease, with the long-term safety and tolerability of injecting autologous NSCs being reported in multiple sclerosis patients.

Second, the main goal of this phase I-II study is to examine the safety of using an allogenic source of MSCs for treatment which would remove individual variability in the MSC secretome observed when autologous stem cells are used as a source of cellular therapy [42]. Although similar clinical trials have sought to examine the safety of a treatment modality, there is scarcity and variability in the aspects assessed and reported by different groups. To improve reporting, our team compiled a tailored, comprehensive questionnaire with which the relevant TEAEs can be assessed according to a grade scale.

Third, the planned dual route of injection is consistent with published data regarding the benefits of intrathecal transplantations. Despite the invasiveness accompanying the procedure, delivering NSCs and MSCs via this route can decrease their chances of being trapped in the lungs and spleen [43]. The intravenous route was used simultaneously to account for the reported benefits and proposed mechanisms of action related to this route [44]. It is worth noting the study by Venkataramana et al [45] in which a single dose of bone marrow–derived MSCs was unilaterally transplanted into the sublateral ventricular zone by stereotaxic surgery into people with PD. Although their study did not report on the effectiveness of the treatment trialed due to the characteristics of the study (limited number of participants and being held in an uncontrolled manner), the results inspired subsequent studies—including our own—to consider cell-based therapy for PD.

Finally, this protocol includes extensive assessment tools for efficacy. Testing the feasibility of combining many measures that reflect the diversity of PD symptoms is of value to future trials regardless of the treatment under study. Although the 6-month follow-up period cannot assess the long-term effects of stem cell therapy, it is being used by many researchers to identify clinical changes in response to different treatment modalities [46].

Another aim of publishing this detailed protocol is to enable criticism and make room for improvement in the design of future trials. For instance, more tools for examining disease, such as neuroimaging and a larger panel of biomarkers, including microRNA, peptides, and metabolites can be added to future trials’ assessment list [46]. Indeed, assessment tools used in this protocol have been carefully combined to cover many aspects of the disease. The APDM Mobility Lab system which is a validated, objective measure, captures gait impairments and postural instability which are amongst the most important motor complications influencing the functional quality of life in PD [47]. In addition, analyzing measured biomarkers during the course of therapy allows for a deeper insight into the changes related to the neurodegenerative and immune aspects of PD. This also provides a more personalized assessment of each participant to account for the lack of consensus on a specific progression marker in PD. Furthermore, the study design amply considers the nonmotor aspects of PD, which are often overlooked, as the burden of nonmotor symptoms, such as hyposmia, sleep disturbances, and cognitive impairments, significantly contribute to the overall poor quality of life among people with PD.

This detailed clinical trial protocol describes the many aspects related to the preparation and administration of stem cells, the recruitment of people with PD, and the safety and efficacy tools used to assess the therapy. It presents a model study design as it contains clear and direct procedures that may help neurologists and stem cell specialists engage in investigator-initiated clinical trials to assess the safety and value of this alternative therapeutic approach. Furthermore, sharing protocols with the scientific community encourages collaboration and the launching of multisite trials that improve participant numbers and result significance. Finally, due to the multifaceted nature of PD symptoms, interdisciplinary collaboration is key to achieving a medical breakthrough in this field.

## Abbreviations

CTC: Cell Therapy Center
MSC: mesenchymal stem cell
NSC: neuronal stem cell
PD: Parkinson Disease
PBS: phosphate-buffered saline
TEAE: treatment-emergent adverse event
UPDRS: Unified Parkinson Disease Rating Scale
WJ-MSC: umbilical cord–derived mesenchymal stem cell

## References

[1] Mhyre TR, Boyd JT, Hamill RW, Maguire-Zeiss KA (2012). Parkinson's disease. Subcell Biochem.

[2] Khan AU, Akram M, Daniyal M, Zainab R (2018). Awareness and current knowledge of Parkinson’s disease: a neurodegenerative disorder. International Journal of Neuroscience.

[3] Poewe W (2008). Non-motor symptoms in Parkinson's disease. Eur J Neurol.

[4] Lee D, Dallapiazza R, De Vloo P, Lozano A (2018). Current surgical treatments for Parkinson's disease and potential therapeutic targets. Neural Regen Res.

[5] Zaoor I, Shafi A, Haq E (2018). Pharmacological treatment of Parkinson disease. Parkinson’s Disease: Pathogenesis and Clinical Aspects.

[6] Zarzycki MZ, Domitrz I (2020). Stimulation-induced side effects after deep brain stimulation - a systematic review. Acta Neuropsychiatr.

[7] Wyse R, Dunbar G, Rossignol J (2014). Use of genetically modified mesenchymal stem cells to treat neurodegenerative diseases. Int J Mol Sci.

[8] Urrutia DN, Caviedes P, Mardones R, Minguell JJ, Vega-Letter AM, Jofre CM (2019). Comparative study of the neural differentiation capacity of mesenchymal stromal cells from different tissue sources: An approach for their use in neural regeneration therapies. PLoS One.

[9] Harris VK, Stark J, Vyshkina T, Blackshear L, Joo G, Stefanova V, Sara G, Sadiq SA (2018). Phase I trial of intrathecal mesenchymal stem cell-derived neural progenitors in progressive multiple sclerosis. EBioMedicine.

[10] Grigoriadis N, Lourbopoulos A, Lagoudaki R, Frischer J, Polyzoidou E, Touloumi O, Simeonidou C, Deretzi G, Kountouras J, Spandou E, Kotta K, Karkavelas G, Tascos N, Lassmann H (2011). Variable behavior and complications of autologous bone marrow mesenchymal stem cells transplanted in experimental autoimmune encephalomyelitis. Exp Neurol.

[11] Dominici M, Le Blanc K, Mueller I, Slaper-Cortenbach I, Marini F, Krause D, Deans R, Keating A, Prockop D, Horwitz E (2006). Minimal criteria for defining multipotent mesenchymal stromal cells. The International Society for Cellular Therapy position statement. Cytotherapy.

[12] Common Terminology Criteria for Adverse Event v3.0 (CTCAE). Cancer Therapy Evaluation Program.

[13] National Cancer Institute Common Terminology Criteriafor Adverse Events (CTCAE) Version 5.0. Cancer Therapy Evaluation Program.

[14] Chotpitayasunondh T, Thisyakorn U, Pancharoen C, Pepin S, Nougarede N (2008). Safety, humoral and cell mediated immune responses to two formulations of an inactivated, split-virion influenza A/H5N1 vaccine in children. PLoS One.

[15] Goetz CG, Nutt JG, Stebbins GT (2008). The Unified Dyskinesia Rating Scale: presentation and clinimetric profile. Mov Disord.

[16] Mancini M, Horak FB (2016). Potential of APDM mobility lab for the monitoring of the progression of Parkinson's disease. Expert Rev Med Devices.

[17] Earhart G, Cavanaugh Jim T, Ellis Terry, Ford Matt P, Foreman K Bo, Dibble Lee (2011). The 9-hole PEG test of upper extremity function: average values, test-retest reliability, and factors contributing to performance in people with Parkinson disease. J Neurol Phys Ther.

[18] El-Gilany AH, Hatata ES, Aayob NS, Refaat R (2012). Validation of the Arabic version of the Berg Balance Scale (A-BBS) among elderly residents in a rural community. Middle East Journal of Age and Ageing.

[19] Elboim-Gabyzon M, Agmon M, Azaiza F (2019). Psychometric properties of the Arabic version of the Activities-Specific Balance Confidence (ABC) scale in ambulatory, community-dwelling, elderly people. CIA.

[20] Halaweh H, Svantesson U, Rosberg S, Willen C (2016). Cross-cultural adaptation, validity and reliability of the Arabic version of the Falls Efficacy Scale-International (FES-I). Med Princ Pract.

[21] Doty RL, Frye RE, Agrawal U (1989). Internal consistency reliability of the fractionated and whole University of Pennsylvania Smell Identification Test. Percept Psychophys.

[22] Jensen MP, Miller L, Fisher L.D (1998). Assessment of pain during medical procedures: a comparison of three scales. Clin J Pain.

[23] Shalash AS, Hamid E, Elrassas HH, Bedair AS, Abushouk AI, Khamis M, Hashim M, Ahmed NS, Ashour S, Elbalkimy M (2018). Non-motor symptoms as predictors of quality of life in Egyptian patients with Parkinson's disease: a cross-sectional study using a culturally adapted 39-Item Parkinson's Disease Questionnaire. Front Neurol.

[24] Fawzi MH, Fawzi MM, Abu-Hindi W (2012). Arabic version of the Major Depression Inventory as a diagnostic tool: reliability and concurrent and discriminant validity. East Mediterr Health J.

[25] Khalil Hanan, Al-Shorman Alham, Alghwiri A, Abdo Nour, El-Salem Khalid, Shalabi Sarah, Aburub Aseel (2019). Cross cultural adaptation and psychometric evaluation of an Arabic version of the modified fatigue impact scale in people with multiple sclerosis. Mult Scler Relat Disord.

[26] Nasreddine Z, Phillips Natalie A, Bédirian Valérie, Charbonneau Simon, Whitehead Victor, Collin Isabelle, Cummings Jeffrey L, Chertkow Howard (2005). The Montreal Cognitive Assessment, MoCA: a brief screening tool for mild cognitive impairment. J Am Geriatr Soc.

[27] Al-Ghatani A, Obonsawin M, Al-Moutaery, C (2010). The Arabic version of the Stroop Test and its equivalency to the English version. Pan Arab Journal of Neurosurgery.

[28] Jaeger J (2018). Digit Symbol Substitution Test: the case for sensitivity over specificity in neuropsychological testing. J Clin Psychopharmacol.

[29] Suleiman KH, Yates BC, Berger AM, Pozehl B, Meza J (2010). Translating the Pittsburgh Sleep Quality Index into Arabic. West J Nurs Res.

[30] Ahmed AE, Fatani A, Al-Harbi A, Al-Shimemeri A, Ali YZ, Baharoon S, Al-Jahdali H (2014). Validation of the Arabic version of the Epworth Sleepiness Scale. JEGH.

[31] BaHammam AS, Al-Aqeel AM, Alhedyani AA, Al-Obaid GI, Al-Owais MM, Olaish AH (2015). The validity and reliability of an Arabic Version of the STOP-Bang Questionnaire for identifying obstructive sleep apnea. Open Respir Med J.

[32] Suleiman K, Yates Bernice C (2011). Translating the insomnia severity index into Arabic. J Nurs Scholarsh.

[33] Dunbar S, Hoffmeyer M (2013). Microsphere-based multiplex immunoassays: development and applications using Luminex xMAP® technology. The Immunoassay Handbook: Theory and applications of ligand binding, ELISA and related techniques.

[34] Waragai M, Sekiyama K, Sekigawa A, Takamatsu Y, Fujita M, Hashimoto M (2010). α-Synuclein and DJ-1 as potential biological fluid biomarkers for Parkinson's Disease. Int J Mol Sci.

[35] Chen-Plotkin AS, Hu WT, Siderowf A, Weintraub D, Goldmann Gross R, Hurtig HI, Xie SX, Arnold SE, Grossman M, Clark CM, Shaw LM, McCluskey L, Elman L, Van Deerlin VM, Lee VM, Soares H, Trojanowski JQ (2011). Plasma epidermal growth factor levels predict cognitive decline in Parkinson disease. Ann Neurol.

[36] Qin X, Zhang S, Cao C, Loh YP, Cheng Y (2016). Aberrations in peripheral inflammatory cytokine levels in Parkinson disease: a systematic review and meta-analysis. JAMA Neurol.

[37] Chen X, Hu Y, Cao Z, Liu Q, Cheng Y (2018). Cerebrospinal fluid inflammatory cytokine aberrations in Alzheimer's disease, Parkinson's disease and amyotrophic lateral sclerosis: a systematic review and meta-analysis. Front Immunol.

[38] Jinfeng L, Yunliang W, Xinshan L, Shanshan W, Chunyang X, Peng X, Xiaopeng Y, Zhixiu X, Honglei Y, Xia C, Haifeng D, Bingzhen C (2016). The effect of MSCs derived from the human umbilical cord transduced by fibroblast growth factor-20 on Parkinson's disease. Stem Cells Int.

[39] Shetty P, Ravindran Geeta, Sarang Shabari, Thakur Anirbhan M, Rao Harinarayana S, Viswanathan Chandra (2009). Clinical grade mesenchymal stem cells transdifferentiated under xenofree conditions alleviates motor deficiencies in a rat model of Parkinson's disease. Cell Biol Int.

[40] Alhattab D, Jamali F, Ali D, Hammad H, Adwan S, Rahmeh R, Samarah O, Salah B, Hamdan M, Awidi A (2019). An insight into the whole transcriptome profile of four tissue-specific human mesenchymal stem cells. Regenerative Medicine.

[41] Harris VK, Vyshkina T, Sadiq SA (2016). Clinical safety of intrathecal administration of mesenchymal stromal cell-derived neural progenitors in multiple sclerosis. Cytotherapy.

[42] Dahbour S, Jamali F, Alhattab D, Al-Radaideh A, Ababneh O, Al-Ryalat N, Al-Bdour M, Hourani B, Msallam M, Rasheed M, Huneiti A, Bahou Y, Tarawneh E, Awidi A (2017). Mesenchymal stem cells and conditioned media in the treatment of multiple sclerosis patients: Clinical, ophthalmological and radiological assessments of safety and efficacy. CNS Neurosci Ther.

[43] Eggenhofer E, Luk F, Dahlke MH, Hoogduijn MJ (2014). The life and fate of mesenchymal stem cells. Front Immunol.

[44] Musiał-Wysocka A, Kot M, Majka M (2019). The pros and cons of mesenchymal stem cell-based therapies. Cell Transplant.

[45] Venkataramana NK, Kumar SK, Balaraju S, Radhakrishnan RC, Bansal A, Dixit A, Rao DK, Das M, Jan M, Gupta PK, Totey SM (2010). Open-labeled study of unilateral autologous bone-marrow-derived mesenchymal stem cell transplantation in Parkinson's disease. Transl Res.

[46] He R, Yan X, Guo J, Xu Q, Tang B, Sun Q (2018). Recent advances in biomarkers for Parkinson's Disease. Front Aging Neurosci.

[47] Mancini M, Horak FB (2016). Potential of APDM mobility lab for the monitoring of the progression of Parkinson’s disease. Expert Review of Medical Devices.

